# The microbial contribution to litter decomposition and plant growth

**DOI:** 10.1111/1758-2229.13205

**Published:** 2023-11-29

**Authors:** Changfeng Zhang, Simone de Pasquale, Kyle Hartman, Claire E. Stanley, Roeland L. Berendsen, Marcel G. A. van der Heijden

**Affiliations:** ^1^ Plant‐Microbe Interactions, Department of Biology, Faculty of Science Utrecht University Utrecht the Netherlands; ^2^ Plant Soil Interactions Division Agroecology and Environment, Agroscope Zürich Switzerland; ^3^ Department of Plant and Microbial Biology University of Zurich Zurich Switzerland

## Abstract

Soil and plant roots are colonized by highly complex and diverse communities of microbes. It has been proposed that bacteria and fungi have synergistic effects on litter decomposition, but experimental evidence supporting this claim is weak. In this study, we manipulated the composition of two microbial kingdoms (Bacteria and Fungi) in experimental microcosms. In microcosms that were inoculated with fungi, litter loss was 47% higher than in microcosms that were not inoculated or only inoculated with bacteria. Combined inoculation with both bacteria and fungi did not significantly enhance decomposition compared with the fungi‐only treatments, and, as such, we found no evidence for complementary effects using our experimental setup. Inoculation with fungi also had a positive impact on plant growth after 4 and 8 weeks (480% and 710% growth stimulation, respectively). After 16 weeks, plant biomass was highest in microcosms where both bacteria and fungi were present pointing to fungal‐bacterial complementarity in stimulating plant growth. Overall, this study suggests that fungi are the main decomposers of plant litter and that the inoculated fungi contribute to plant growth in our experimental system.

## Graphical abstract

The presence of fungi and bacteria was manipulated in microcosms to investigate their effect on litter decomposition and plant growth. Fungal inoculation resulted in a 47% higher rate of litter decomposition compared to microcosms without fungal inoculation. The contribution of bacterial inoculation to litter decomposition was small. Additionally, microcosms that were inoculated both with fungi and bacteria exhibited the highest plant biomass. Graph Created with BioRender.com

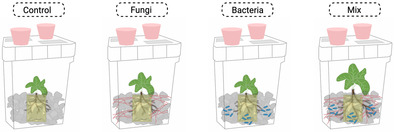

## INTRODUCTION

Soil microbes are highly abundant and represent the “unseen majority” on earth, providing one of the largest pools of genetic diversity (Anthony et al., 2023; Bardgett & van der Putten, 2014; Roesch et al., 2007; Whitman et al., 1998). Moreover, soil communities are fundamental for maintaining important ecosystem processes (Banerjee & van der Heijden, 2023; Wagg et al., 2014; Wagg et al., 2019). Bacteria and fungi are dominant members of soil microbial communities, interacting not only with one another but also with plant roots, as they share the same habitats. These multi‐kingdom interactions vary, and synergistic effects on plant growth and health have been repeatedly observed (Etesami et al., 2021; van der Heijden et al., 2015).

A wide range of studies has analysed the composition and diversity of microbes colonizing the soil and inhabiting plant roots (Fierer, 2017; Fitzpatrick et al., 2018; Gaiero et al., 2013; Lareen et al., 2016; Lundberg et al., 2012). While much progress has been made to catalogue such microbial communities, much less is known about the actual functions of individual microbes and microbial communities. Some groups of microbes have been widely investigated (e.g., nitrogen‐fixing rhizobia bacteria, plant growth‐promoting bacteria, a wide range of mycorrhizal fungi and microbial pathogens) (Garrido‐Oter et al., 2018; Pieterse et al., 2021; van der Heijden et al., 2015; Xin & He, 2013), but the function of the majority of microbes, including a wide range of rhizosphere‐inhabiting microbes is still poorly understood. Here, we focus on microbes isolated from *Trifolium* roots (excluding well‐known nitrogen‐fixing bacteria and mycorrhizal fungi), and we test the impact of these bacteria and fungi on litter decomposition and plant growth.

Although a range of studies linked the decomposition of plant litter to the bacterial and fungal communities that colonize litter (Mei et al., 2022; Purahong et al., 2016; Zheng et al., 2021), the relative contribution of bacteria, fungi and their interactions to litter decomposition are poorly understood. Only very few studies have experimentally manipulated the presence and abundance of bacteria and fungi to assess their roles in litter decomposition (Wagg et al., 2014). Fungi exude a range of extracellular enzymes (Romaní et al., 2006; Schneider et al., 2010; Schneider et al., 2012), and based on metaproteomics, it was proposed that fungi contribute much more to decomposition and C loss than bacteria (Chen et al., 2021; Pascoal & Cássio, 2004). This implies that fungi are the main drivers of litter decomposition. However, bacteria do appear to influence the litter decomposition process. Some studies suggest that bacteria complement fungi when decomposing litter (Güsewell & Gessner, 2009; Zhao et al., 2021) and that certain bacteria contribute to the production of extracellular degrading enzymes in the later stages of decomposition (Kirby, 2005). For instance, *Betaproteobacteria* and *Dothideomycetes* showed higher litter degradation capability in larch litter (Sauvadet et al., 2019). Moreover, based on network analyses, the bacteria from the genus *Chryseobacterium* have been identified as one of the keystone taxa in litter decomposition processes (Zheng et al., 2021). In contrast, other studies found much lower litter degradation activities in bacterial communities (Pascoal & Cássio, 2004; Schneider et al., 2010; Schneider et al., 2012).

To investigate the relative contributions of fungi and bacteria to plant growth and litter decomposition, experimental microcosms filled with sterilized soil and plant litter and planted with the herb *Prunella vulgaris* were inoculated with either a synthetic community of (1) bacteria (41 strains), (2) fungi (35 strains), (3) bacteria and fungi together, or (4) a negative control that did not receive an inoculum. The effects of these treatments on plant growth and litter decomposition were assessed every 4 weeks for 16 weeks, and we subsequently used amplicon sequencing to verify which bacterial and fungal taxa established and colonized the plant litter and plant roots. The plant species and microbial taxa used for this experiment all co‐occur at one field site.

## EXPERIMENTAL PROCEDURES

### Microcosm construction and preparation

Magenta GA‐7 boxes were used as experimental microcosms and modified after (Hartman et al., 2017). The lids of the boxes contained two holes (⌀ 1.5 cm) and were sealed with gas‐permeable foil for air exchange. The boxes were filled with 90 g calcined clay, marketed as Oil‐Dri (Damolin GmbH, Oberhausen, Germany). Two litter bags were buried in the substrate in a back‐to‐back position into magenta boxes. Each litter bag contained 0.3 g of dried *Lolium multiflorium* litter. The litter bags are made of nylon mesh with a pore size of 30 μm, which prevents plant roots from accessing them. During autoclaving and for short‐term storage, the magenta boxes (covered with aluminium foil) and lids were placed inside two autoclavable bags, thus providing a double layer of protection and preventing accidental contamination in case one bag was later damaged during the experimental setup. The microcosms and lids were autoclaved twice for 99 min at 121°C. We plated autoclaved soil substrate onto agar plates and confirmed that the autoclaving protocol successfully deactivated all microbes. The plant species used in the microcosms (*P. vulgaris*), the plant species used to produce litter (*Lolium multiflorum*), and the plant species used as a source to isolate the microbes from the field (*Trifolium pratense*) all co‐occur at the field site (the FArming Systems and Tillage experiment, hereafter the FAST experiment, 47°26 ′20″ N 8°31′40″ E). The FAST experiment is an arable farming systems trial with grassland strips established in 2009 (see Wittwer et al., 2021 for further details about the location, soil type and climate). Note, however, that we made use of a model system and our results are not comparable with the situation in the field. We grew the plants in sterilized substrate in a growth chamber and the number of microbes used to inoculate the microcosms is much lower than the number usually present in the field.

### Seed germination for planting

*P. vulgaris* has been regularly used as a model plant in ecological and evolutionary research (Miller et al., 1994; Qu & Widrlechner, 2011; Streitwolf‐Engel et al., 2001; Winn, 1988; Winn & Gross, 1993), and its small size fits well for gnotobiotic system construction and manipulation in small microcosms. *P. vulgaris* seeds were surface sterilized for 5 min in 70% EtOH, followed by 5 min in 5% NaClO and rinsed three times with sterile distilled water. The seeds were sown on 1/2 Murashige and Skoog basal medium (Sigma Aldrich, St. Louis, MO, USA) supplemented with 1% sucrose. A maximum of 10 seeds were sown on one plate to prevent cross‐contamination. After 2 days of stratification at 4°C, the plates were transferred to a climate chamber (Sanyo MLR‐352H; Panasonic, Osaka, Japan) under controlled conditions (25°C, 16 h light 100 μE/m^2^/s; 16°C 8 h, dark). Seedlings with roots of approximately ~0.5 cm length that were free of visible contamination, but potentially containing endophytes were selected for planting in the microcosms.

### Microbial community creation

We made use of a previously published bacteria collection isolated from naturally collected and climate chamber cultivated roots of *T. pratense* (Hartman et al., 2017), collected from the field site, the Farming Systems and Tillage experiment, an arable farming systems trial with grassland strips established in 2009 (Wittwer et al., 2021). Fungal isolates were isolated from *T. pratense* root fragments described in the supplementary method. The isolates were sequenced using the primer pair 27F and 1401R (Nübel et al., 1996) for bacteria and the primer pair ITS5 and ITS4 (White et al., 1990) for fungi. The microbial isolates were clustered into operational taxonomic units (OTU) at >97% sequence similarity and a total of 41 bacterial and 35 fungal OTUs were detected (e.g., for most OTUs several strains were detected). For each OTU, we randomly selected one bacterial or one fungal strain for inoculation of the microcosms (Table S1).

### Preparation of the microbial treatment inocula

Four microbial community treatments (*Control*, *Bacteria*, *Fungi* and *Mix*) were used in our study. We inoculated pure cultures of 41 bacterial strains in the Bacteria (only) treatment. The Fungi (only) treatment received pure cultures of 35 fungal strains. The Mix treatment was inoculated with 41 bacteria and 35 fungi. The Control treatment was not inoculated with any microbes and received sterilized agar plugs to standardize all treatments and to ensure that the addition of microbial inocula did not influence soil nutrient availability. These bacteria and fungi were isolated from the roots of *Trifolium pratense* collected from the long‐term FAST experiment (Wittwer et al., 2017; Wittwer et al., 2021). An earlier study demonstrated that the inoculated bacteria and fungi not only colonize roots but are also abundant in the arable and grassland soil at this location (Hartman et al., 2017; Hartman et al., 2018).

The selected bacteria and fungi were revived from glycerol stocks stored at −80°C by plating on Flour Medium agar (FMA; (Coombs & Franco, 2003) and Mathur's Medium agar (MMA; (Freeman & Katan, 1997), respectively. The bacteria plates were cultured at 28°C for one to 2 weeks. The fast growers were stored at 4°C and subsequently, the fast and slow growers were subcultured at the same period. We scraped off the bacteria colonies of each strain and mixed them with 100 μL sterile distilled water. Subsequently, 100 μL of each bacteria suspension was pipetted onto an FMA plate, and the mixture was spread around the plate with a flamed glass spreader. The plates were incubated at 28°C for up to 2 weeks, or until bacteria colonies had covered the entire plate.

The fungi plates were cultured at 26°C for one to 2 weeks to ensure enough growing time for slower‐growing fungi. Fungi were sub‐cultured by taking agar plugs (ø 5 mm) from each strain and transferring them to a new MMA plate. The sub‐cultured plates were incubated at 26°C for up to 2 weeks, or until fungi hyphae covered the entire plate. Faster‐growing isolates were stored at 4°C until use. Three replicate plates per bacterial and fungal isolate were plated to ensure enough biomass for inoculum creation.

The microbial inoculum for each microcosm was created independently. One agar plug (5 mm ø) of each strain was added into a sterile 50 mL Falcon tube for each microcosm. Therefore, 41 bacteria plugs were added per tube in the bacteria treatment. In addition, the bacteria treatment received 35 sterile MMA plugs to ensure equal nutrient additions across all treatments. Similarly, the inoculum for the fungi treatment included 35 fungi plugs and 41 sterile FMA plugs for nutrient adjustment. The Mix treatment contained 35 bacteria plugs and 41 fungi plugs, and each Control treatment microcosm was inoculated with 35 sterile FMA plugs and 41 sterile MMA plugs.

Subsequently, 20 mL of sterile 15% Hoagland solution (Table S2) was added to the tube and the contents were blended with a sterile laboratory blender (Polytron, Kinematica, Lucerne, Switzerland; setting 3 for 30 s). The head of the blender was surface sterilized by submersing in 70% ethanol for 10 min, and then in 5% sodium hypochlorite for 20 min. The head of the blender was then rinsed three times with sterile distilled water. The blender was surface sterilized between inoculum preparation of the different treatments to prevent cross‐contamination. The efficiency of the surface sterilization procedure was verified by plating 100 μL of the water used for rinsing the blender on FMA and MMA and checking for microbial growth. After blending the plugs and Hoagland solution mixture into a slurry, the slurry volume in each tube was adjusted to 45 mL with 15% Hoagland solution to create the inoculum for each microcosm.

### Microcosm assembly

The inoculation of the microcosms was performed in a sterile laminar flow cabinet. 45 mL of inoculum was poured evenly over the surface of the substrate in the microcosm, followed by another 45 mL of sterile 15% Hoagland solution to ensure enough water and nutrients for plant growth. Two pre‐germinated seedlings were sown in the substrate with a sterile spatula. The microcosms were closed with the lids and then sealed with parafilm. The microcosms were randomly distributed across the shelves of the climate chamber (25°C, 16 h light, 16°C, 8 h dark; 70% relative humidity). Every week, the microcosms were randomly reallocated to new positions in the climate chamber to minimize any effects of environmental variability.

### Harvest

A total of 96 microcosms were set up, and microcosms were harvested after 4, 8, 12 and 16 weeks (4 treatments * 4 time points * 6 replicates = 96 microcosms). Harvesting was performed in a sterile laminar flow cabinet. Above‐ground plant biomass was cut using a sterile scalpel, dried in paper bags for 48 h at 60°C and weighed. The plant roots that were loosely attached to calcined clay were shaken gently and collected using sterile tweezers, placed into 50 mL tubes and immediately frozen at −20°C. The litter bags were removed by sterile tweezers. One litter bag from each microcosm was rinsed with distilled water to remove substrate particles, dried in paper envelopes at 60°C for 48 h and weighed. The other litter bag from each microcosm was placed in a sterile 50 mL tube and stored at −20°C. The remaining growth substrate was collected in a 50 mL tube and stored at −20°C.

### Quantification of active microorganisms in microcosms by serial dilution

At the 8th‐week and 16th‐week harvests, 1 g of substrate was sampled from each microcosm and serially diluted on FMA and MMA plates to quantify the active bacteria and fungi, respectively. The substrate was mixed with sterile 0.9% saline water, vortexed for 1 min and serially diluted to 10^−6^. For each microcosm, 50 μL of the 10^−4^, 10^−5^ and 10^−6^ dilutions was spread on FMA and MMA plates separately. The colony forming units (CFU) were calculated after 3 days until 7 days.

### Litter and root microbiome profiling

Because our root sampling method did not discriminate between the rhizoplane (root surface) or the endosphere (root interior) compartments, we refer generally to the sampled unit as “root microbiome”. After 8 weeks, the litter and root microbiomes were characterized by conducting 16S rRNA gene and ITS amplicon sequencing. Litter and root samples were lyophilized for 48 h. DNA was extracted from litter and root samples using the NucleoSpin Soil DNA extraction kit (Machery‐Nagel GmbH & Co. KG, Düren, Germany) according to the manufacturer's instructions. Extracted DNA was quantified using Qubit® (1.0) Fluorometer and the Tapestation (Agilent Technologies, Santa Clara, CA USA).

### 16S and ITS PCR and library preparation

We amplified the V3 and V4 regions of the 16S rRNA gene using PCR primers 341F and 806R (Takahashi et al., 2014), targeting a single amplicon of approximately ~460 bp. The concentration of DNA samples was diluted to 5 ng/μl and used in a two‐step PCR amplification protocol. The first PCR reaction was processed on a thermocycler (Hybaid, Ashford, UK) using the KAPA HiFi HotStart ReadyMix (F. Hoffmann‐La Roche AG, Basel, Switzerland) PCR system with the cycling conditions in Table S3. Each sample was amplified in a 12 μL reaction volume containing 1.5 μL of 5 ng/μl DNA template, 7.5 μL KAPA, 1.5 μL of 2 μM concentrated forward and reverse primers. The primers were adapted with a 0–7 base heterogeneity spacer to enhance sequence diversity (Wu et al., 2015). The resulting PCR products were purified using AMPure XP beads (Beckman Coulter, High Wycombe, UK) according to the manufacturer's instructions. The purified PCR products were then used as template DNA in the second PCR (Table S3). Each sample was amplified in a 25 μL reaction volume containing 2.5 μL DNA template, 12.5 μL KAPA, 2.5 μL 2 μM forward and reverse primers and 5 μL MilliQ‐purified water. The primers were adapted with an error‐tolerant 6‐mer barcode to allow pooling of the multiplexed PCR products. The resulting PCR products were then cleaned up using AMPure XP beads. Afterward, we loaded 5 μL of each sample on an agarose gel to check for correct amplicon size and used a Qubit® (1.0) Fluorometer to quantify the DNA concentration in each sample. Each library of 5 μL 4 nM DNA was pooled together.

For ITS amplicon library preparation, we targeted the ITS1 region yielding a ~ 300 bp amplicon using primers ITS1F (Gardes & Bruns, 1993) and ITS2 (Op De Beeck et al., 2014). We prepared the ITS library following the same protocol as for the 16S rRNA gene amplification. In short, the diluted 5 ng/μl DNA was first amplified in a 15 μL reaction volume containing 2.5 μL 1 μM forward and reverse primer, 7.5 μL KAPA and 10 ng DNA template. The PCR products were purified using AMPure XP beads and the resulting DNA was used as a template in the second PCR using the same conditions for the 16S mentioned above. Both PCR cycling conditions are shown in Table S3. The PCR products were cleaned up with AMPure XP beads and DNA concentration was quantified by Qubit® (1.0) Fluorometer. Equal PCR product amounts (5 μL 4 nM) were pooled together. The 16S library and ITS library were mixed and sequenced on Illumina MiSeq Sequencer (Illumina, San Diego, USA) using a paired‐end 300 bp V3 kit at Utrecht Sequencing Facility (www.useq.nl).

### Sequence data processing

We employed the Qiime2 environment (version 2019.07, https://qiime2.org/) for sequence processing. The quality of the paired‐end sequences was assessed using the *Demux* plugin. Primers of imported sequences were removed via Cutadapt (Martin, 2011). The paired‐end sequences were merged using the vsearch join‐pairs script, allowing the joining of staggered read pairs to retain as many sequences as possible (Rognes et al., 2016). Deblur (Amir et al., 2017) was used to filter, denoise sequences, trim sequences to a common length (16S: 269 bp, ITS: 200 bp) and remove chimeras. The filtered sequences were subsequently clustered to OTUs (Operational taxonomic units) at 97% sequence similarity. 16S and ITS OTUs were taxonomically annotated using a pre‐trained naive Bayes classifier (Werner et al., 2012) against the Greengenes reference database (release 13_5, 99% OTUs) (McDonald et al., 2012) and the UNITE (v8, 04.02.2020, 99% OTUs) (Abarenkov et al., 2010) databases, respectively. From this taxonomic assignment, 16S OTUs annotated as mitochondria and chloroplast were removed. The denoised sequences of the bacterial community and fungal communities were then rarefied to 1000 and 10,000 sequences per sample (Figure S1A,B), respectively. To preserve the low sequence depth of the fungal community in the Control and the Bacteria treatments, we also show the not‐rarefied fungal OTU richness in Figure S2. The raw sequencing data were deposited at the European Nucleotide Archive (http://www.ebi.ac.uk/ena) by the study accession PRJEB54741.

### Rediscovery of inoculated strains in the microcosms

To identify which of the inoculated bacterial and fungal strains established in the microcosms, we mapped the sequences of the inoculated microbes to corresponding OTU sequences of the community profiling. The bacterial sequences were aligned and trimmed based on their 16S rRNA v3‐v4 region ClustalW (Thompson et al., 2003) in MEGA X (Sudhir et al., 2018). The trimmed 16S rRNA sequences and the untrimmed ITS sequences were imported to Qiime2 and used as query sequences to map with clustered OTUs using the “quality‐control exclude‐seqs” script (Camacho et al., 2009) at 100% sequence similarity (Tables S4 and S5).

### Statistical analyses

All statistical analyses were conducted in R version 4.0.2 (R Core Team, 2013). Differences in community composition between the bacterial and fungal communities in the different microbial treatment and sample types were tested by pairwise permutational analysis of variance (PERMANOVA) on Bray‐Curtis dissimilarities using the *adonis* function in the *vegan* package (Oksanen et al., 2019) with 999 permutations. CFU numbers were assessed for variation among treatments by Kruskal–Wallis followed by Dunn's test. Plant productivity, litter loss percentage and the observed OTU were assessed for variation among treatments by ANOVA and followed by a Tukey HSD test. The observed OTUs' variation within treatments between sample types was determined by a t‐test. Two‐way ANOVA was used to test the effect of microbial treatment on litter decomposition over time. All bioinformatic files generated by qiime2 were imported to R by qiime2R package (Bisanz, 2018). The bacterial and fungal OTUs were rarefied and the observed OTUs were plotted at each rarefaction level using phyloseq package (McMurdie & Holmes, 2013) and ggplot2 (Wickham, 2009). The OTUs that positively associated with one or a combination of microbial treatments were determined by a correlation‐based indicator species analysis with the R package *indicspecies* (Cáceres & Legendre, 2009). The observed OTUs were calculated in qiime2 by the diversity core‐metrics‐phylogenetic script, and the differences across microbial treatments and sample types were determined by Two‐way ANOVA in R.

## RESULTS

In this study, we assessed the effects of the microbial treatments on plant growth and litter decomposition every 4 weeks for 16 weeks and subsequently, we used amplicon sequencing to characterize the plant litter and root microbiome.

### More active bacteria and fungi detected in microbe‐inoculated treatments

**FIGURE 1:**
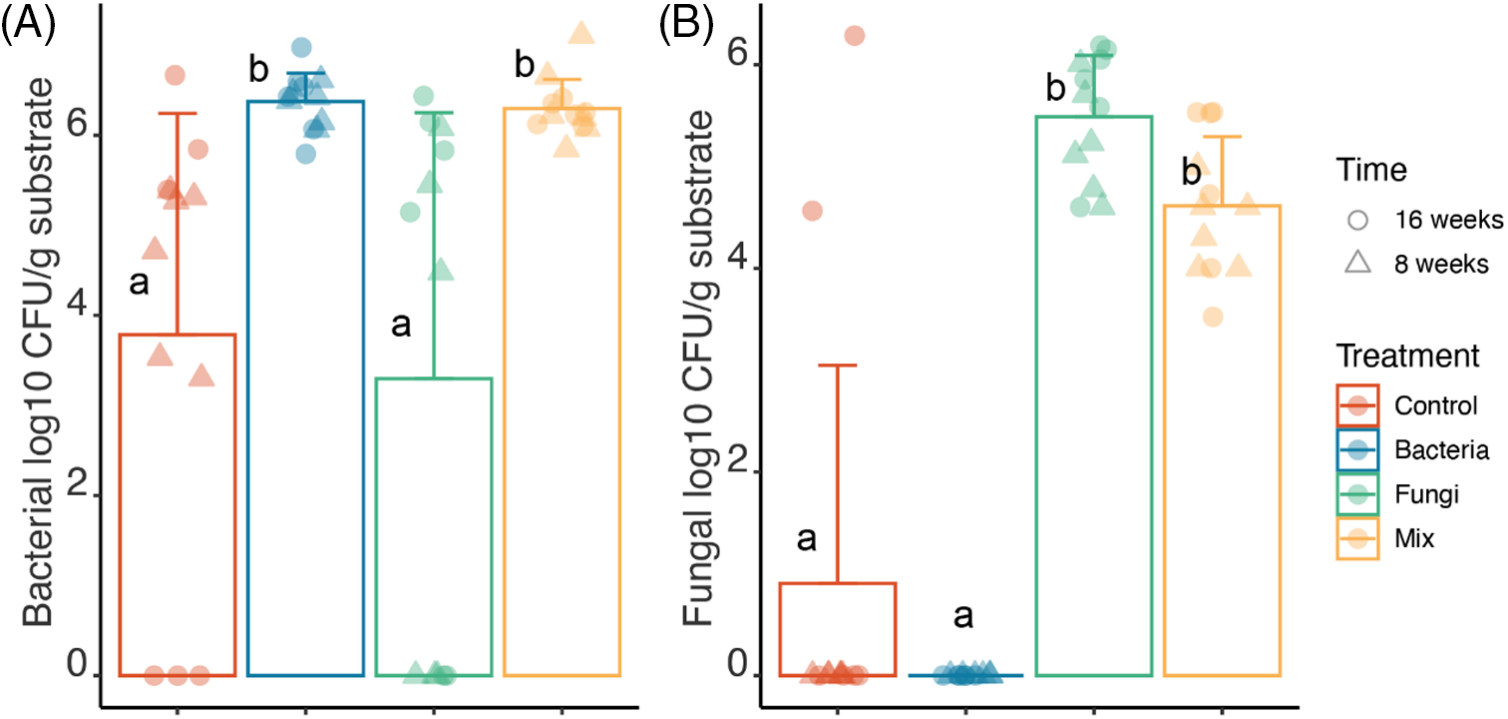
Higher CFU numbers are present in bacteria and fungi‐inoculated treatments. (A) Number of bacterial CFUs across four microbial treatments. (B) Number of fungal CFUs across microbial treatments. Harvest time points are indicated by different symbols. CFU numbers are Log10 transformed. Two samples in the Control treatment that were contaminated with fungal CFUs were excluded from further analysis. treatments are indicated by colours. The Kruskal Wallis test and Dunn's post‐hoc test (*p* < 0.05, Table S6) were performed to determine the significant differences between microbial treatments that are indicated by different letters in the boxplots.

Autoclaved calcined clay was plated on agar plates after 1 week to confirm that autoclaving had successfully sterilized the microcosm system (Data not shown). We did not detect the growth of microbes on these agar plates. The results suggested that the autoclaved substrate was completely free of microbes. To determine whether active microbes survived in the microcosms after 8 and 16 weeks of plant growth, we plated serial dilutions of subsamples of the substrate on an agar‐solidified medium and counted the colony‐forming units (CFUs; Figure 1A). The abundance of bacterial CFUs was significantly higher (on average 5.1–5.7 times) in treatments inoculated with bacteria (Bacteria and Mix) compared with treatments not inoculated with bacteria (Control and Fungi). However, we also noted bacterial CFUs in the Control and Fungi treatments, indicating some bacterial contamination (e.g., from plant endophytes or introduced during the experiment) had occurred during the experiment. Fungal CFU counts were significantly higher in fungi‐inoculated treatments (Fungi and Mix) than in the non‐fungi‐inoculated treatments (Control and Bacteria). Overall, fungal CFU counts in Control and Bacteria were below detection limits, except for two replicates in the Control (Figure 1B).

### Bacteria and fungi inoculated treatments forming specific rhizosphere and litter consortium

We employed 16S rRNA gene and ITS amplicon sequencing to profile the diversity and community composition of bacterial and fungal communities colonizing litter and root samples and to verify which inoculated bacteria and fungi were established in the microcosms.

**FIGURE 2:**
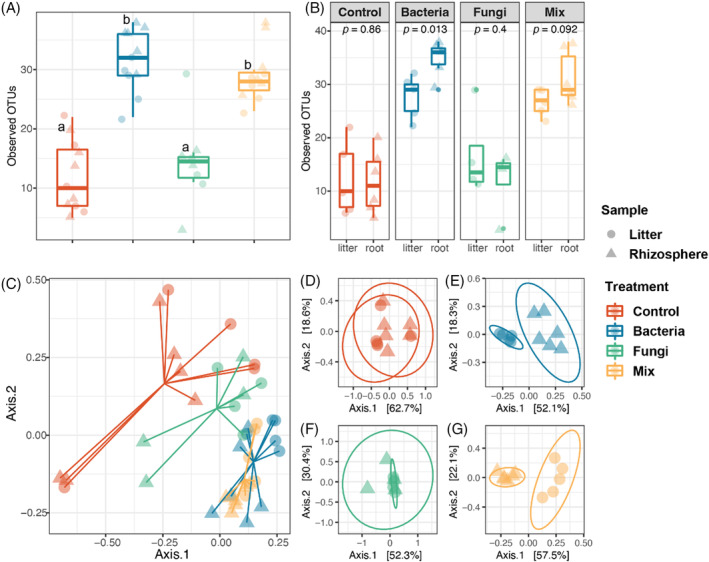
Bacterial community diversity across treatments. (A) Bacterial OTU richness across all treatments. Significance differences between treatments are indicated with letters (*p* < 0.05, ANOVA and Tukey's Honest HSD test). (B) The comparison of bacterial OTU richness between sample types in each microbial treatment. The significance levels were determined by *t* test. (C) The bacterial community PCoA is based on Bray‐Curtis distances. (D) The bacterial communities of Control treatment PCoA are based on Bray‐Curtis distances (PERMANOVA by sample type, pseudo‐F = 1.268, R^2^ = 0.123, *p* value = 0.234). (E) The bacterial communities of Bacteria treatment PCoA based on Bray‐Curtis distances (PERMANOVA by sample type, pseudo‐F = 7.980, R^2^ = 0.469, *p* value = 0.003). (F) The bacterial communities of Fungi treatment PCoA based on Bray–Curtis distances (PERMANOVA by sample type, pseudo‐F = 0.974, R^2^ = 0.139, *p* value = 0.463). (G) The bacterial communities of mix treatment PCoA are based on Bray‐Curtis distances (PERMANOVA by sample type, pseudo‐F = 10.558, R^2^ = 0.539, *p* value = 0.005). Samples are colour‐coded by treatments. The sample types are indicated by different symbols. The results of two‐way ANOVA of the effects of the treatments and sample types are shown in Table S9.

Bacterial inoculation significantly increased bacterial OTU (bOTU) richness, which was on average 2.3–2.6 times higher in the Bacteria and Mix treatments compared with the Control or the Fungi treatment (Figure 2A; Table S7). In the Bacteria treatments, the bacterial richness of the root samples was significantly higher (11.6%) than in the litter samples (Figure 2B). For a more in‐depth analysis of differences in the structure of the bacterial communities in the different treatments, principal coordinate analysis (PCoA) and pairwise PERMANOVA on Bray–Curtis dissimilarities were performed. We noted a clear separation between bacterial‐inoculated treatments and non‐bacterial‐inoculated treatments in the ordination space (Figure 2C) and pairwise PERMANOVA testing confirmed the significant differences between microbial treatments (Table S8). Moreover, litter and root bacterial communities separated on Axis 1 when they received the same bacterial inoculum, but not when the microcosms did not receive any bacterial inoculum (Figure 2D–G).

**FIGURE 3:**
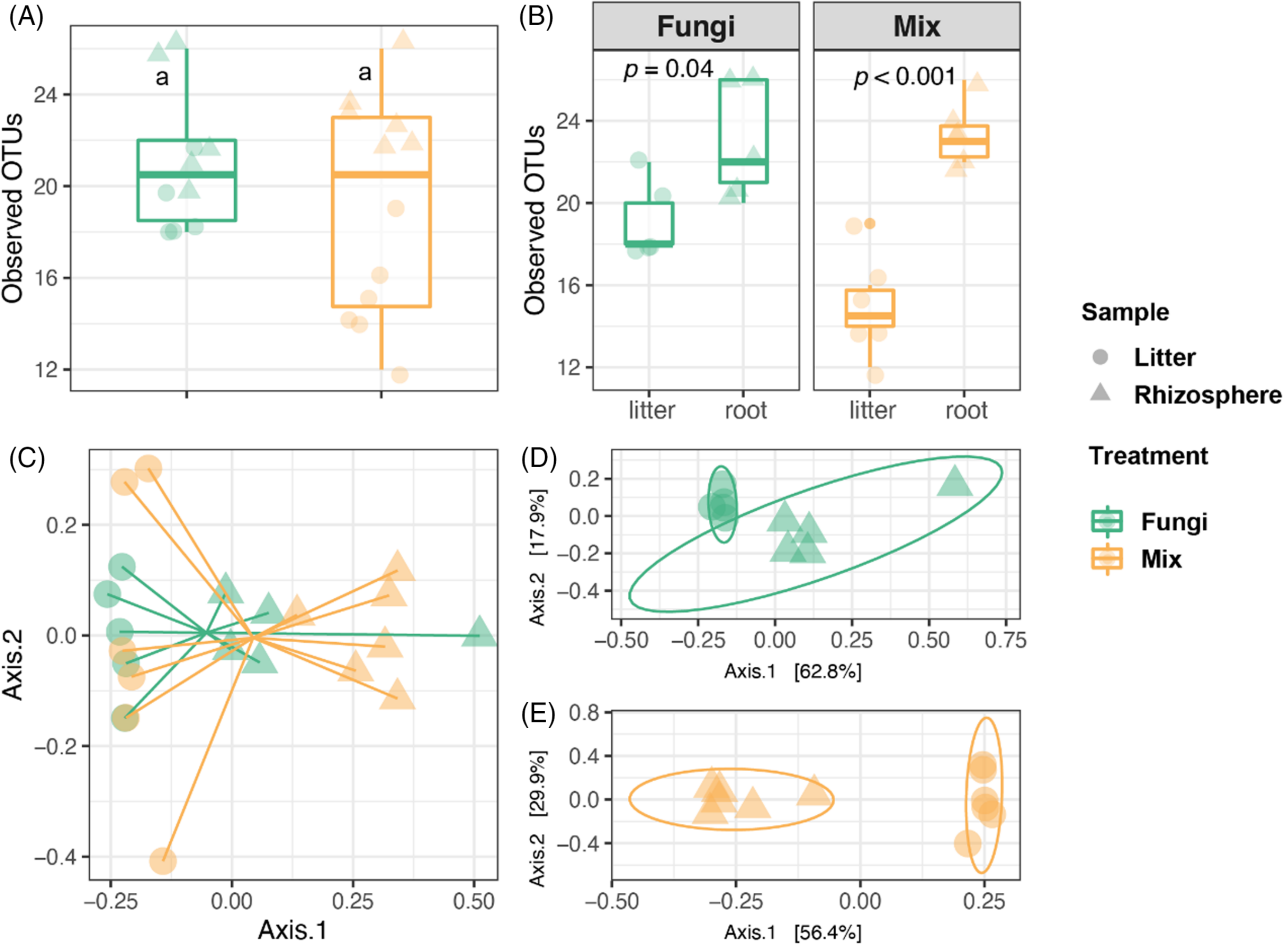
Fungal community diversity across treatments. (A) Fungal OTU richness in Fungi and Mix. Significance differences between treatments are indicated with letters (*p* < 0.05, ANOVA and Tukey's Honest HSD test). (B) The comparison of fungal OTU richness between sample types in each microbial treatment. The significance levels were determined by *t* test. (C) The fungal community PCoA is based on Bray–Curtis distances. (D) The fungal community of Fungi treatment PCoA based on Bray–Curtis distances (PERMANOVA by sample type, pseudo‐F = 5.879, R^2^ = 0.424, *p* value = 0.009). (E) The fungal community of mix treatment PCoA based on Bray–Curtis distances (PERMANOVA by sample type, pseudo‐F = 11.752, R^2^ = 0.540, *p* value = 0.001). Samples are colour‐coded by treatments. Due to low fungal sequence depth in the non‐fungal (Control and Bacteria) treatments (shown in Figure S1b), no values are shown for these two treatments. The sample types are indicated by different symbols. The results of two‐way ANOVA of the effects of the treatments and sample types are shown in Table S9.

Similarly, OTU richness in fungal inoculated treatments was higher compared with the Control or Bacteria (Figure S2, Table S7). We detected very few fungal sequences in non‐fungal (Control and Bacteria) microcosms (Figure S1b), corroborating the serial‐dilution results in which fungal CFUs were lower than the smallest dilution plated (10^−4^) in the large majority of microcosms where no fungi were inoculated (Figure 1B). Fungal OTU (fOTU) richness did not differ between the Fungi and Mix (Figure 3A) and was significantly higher in root samples than in the litter samples (Figure 3B). The PCoA and PERMANOVA show no significant difference between Fungi and Mix (Figure 3C, Table S10). Similar to bacterial communities, the litter and root fungal communities separated on Axis 1 when they received the same fungal inoculum (Figure 3D,E). Together with bacterial data, the separation of litter and root microbial communities suggests that each ecological niche has its preferred microbiome.

### Rediscovering fungal and bacterial inoculates in the microbial communities

**FIGURE 4:**
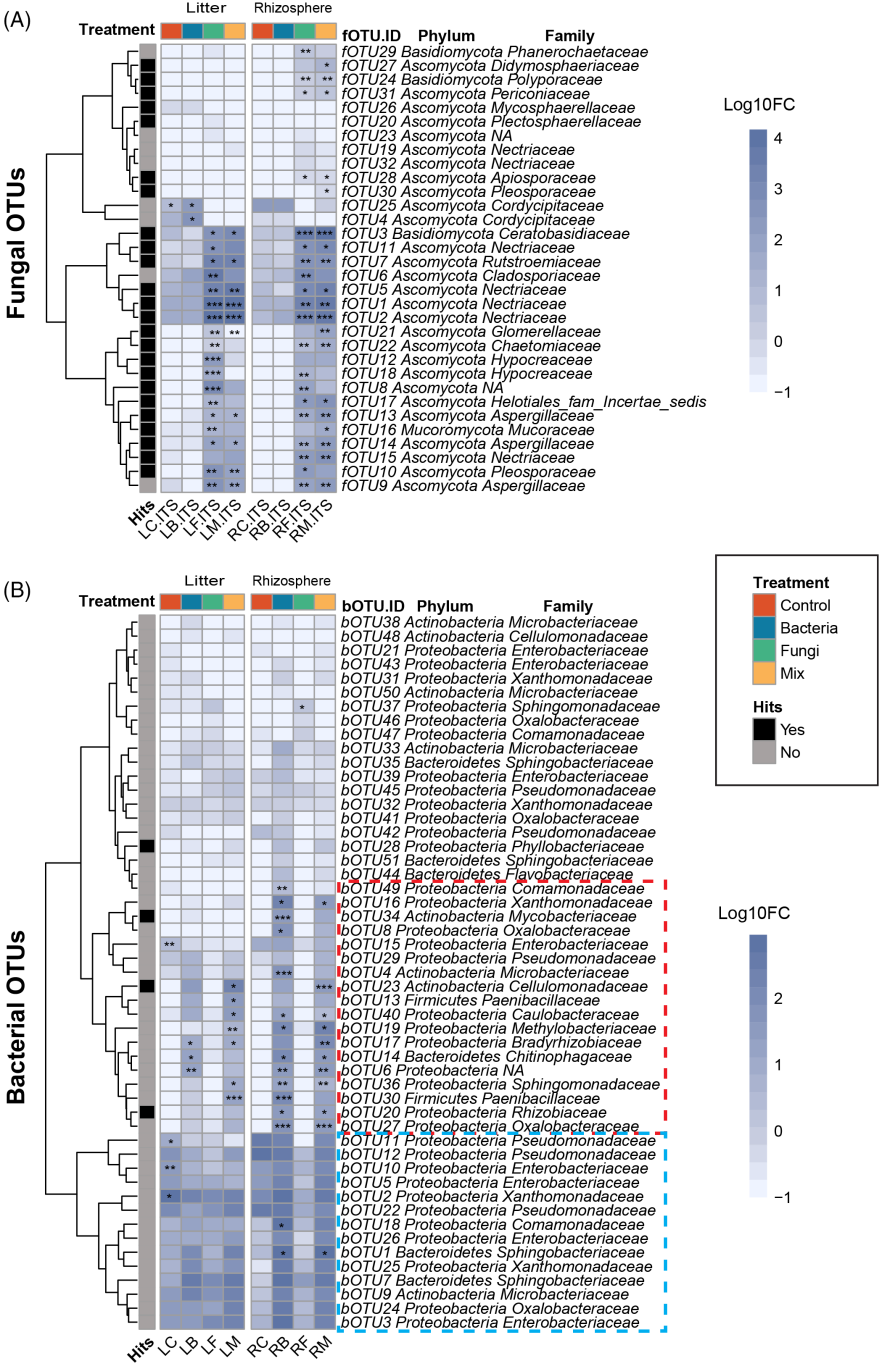
Relative abundance of bacterial and fungal OTUs in litter (L) and root (R) samples inoculated with fungi (F), bacteria (B), fungi and bacteria (M), or non‐inoculated controls (C). Black boxes (Hits) refer to OTU sequences that are similar to inoculated bacterial or fungal while grey boxes (Non‐hits) are OTUs that are not similar to the inoculated bacteria and fungi. The asterisk in the heatmap cells indicates OTUs that are significantly and positively correlated to one or more treatments (p* < 0.05, p** < 0.01, p*** < 0.001). (a) Litter and root fungal OTUs. (b) Litter and rhizosphere bacterial OTUs. Only OTUs present in at least 3 samples are shown here. The red dashed frame indicates the abundant bOTUs of bacterial inoculated treatments of root microbiome. The blue dashed frame indicates that bOTUs are abundant in all treatments of litter microbiome. The dendrogram is based on hierarchical clustering. Litter, rhizosphere and microbial treatments are represented by different colours.

In the next step, we determined which of the inoculated fungal and bacterial taxa established and could be detected in the microcosms. For this, the sequences of the 35 inoculated fungi were mapped to the representative OTU sequences of the fungal community profiles at 100% sequence similarity. Similarly, the 41 sequences of the inoculated bacteria were mapped to the bOTU sequences (Figure 4). In all root and litter samples, we detected 51 bOTUs and 32 fOTUs (Tables S4 and S5). The 35 inoculated fungal sequences matched 23 fOTUs in the community profile. Thus, 65% of the inoculated fungi were established and could be rediscovered. The most abundant fOTUs belonged to the phylum *Ascomycota* (24 fOTUs). One fOTU belonged to the Basidiomycota, and one fOTU belonged to *Mucoromycota*. These taxa were nearly exclusively present in the Fungi inoculated treatments (Fungi and Mix) (Figure 4). In litter samples, 10 fOTUs were abundant in both fungi‐inoculated treatments (Figure 4A). In the root samples, more fungal taxa were detected, with 15 fOTUs enriched in fungi‐inoculated treatments (Figure 4A).

In the bacterial community, 4 (bOTU 20, 23, 28, 34) out of the 41 inoculated bacteria were rediscovered in the community profiles when using a 100% sequence similarity level between inoculated bacteria and bacteria detected in the treatments where bacteria were inoculated (data for 97% sequence similarity are shown in Figure S3A,B). 18 bOTUs were significantly more relatively abundant in the inoculated treatments compared with the control treatment, and this was clear for the root microbiome (bOTUs in the red dashed frame in Figure. 4B). In the litter samples, we noted that one *Bradyrhizobiaceae* (bOTU 17) was abundant in both bacteria inoculated treatments. However, we also observed four bOTUs (bOTU 38, 33, 28, 44) that were present in low abundance in all bacterial inoculated treatments. 14 bOTUs (bOTUs in the blue dashed frame in Figure. 4B) were generally found in all treatments. These bOTUs belonged to *Proteobacteria* (11 bOTUs), *Bacteroidetes* (2 bOTUs) and *Actinobacteria* (1 bOTU).

### Fungi as main decomposers

**FIGURE 5:**
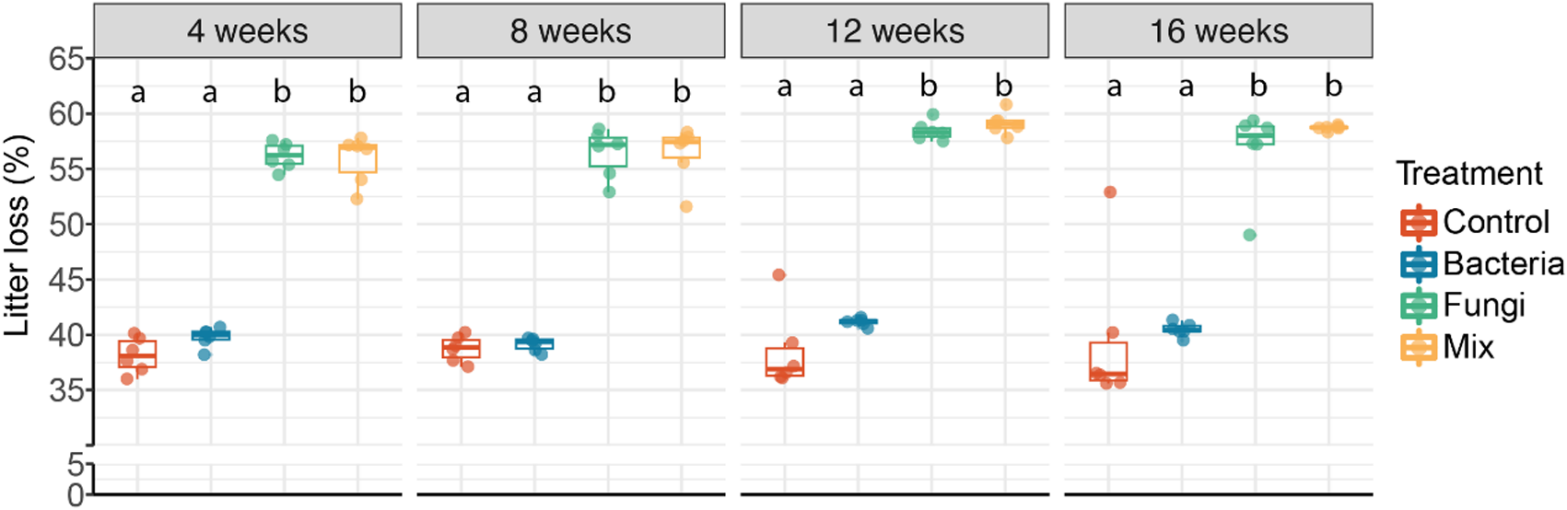
Percentage of litter loss across microbial treatments with time. Significance differences are represented by letters (*p* < 0.05, one‐way ANOVA and Tukey's HSD test).

To assess the relative contribution of bacteria and fungi to litter decomposition, we investigated litter loss in litter‐filled microcosms. The litter loss was higher when fungi were present (Figure 5). This was true for both the Fungi and Mix treatments. The Control and Bacteria treatments, which did not have fungi, had lower litter loss amounts. This difference was significant at every time point. Litter loss in the Bacteria treatment did not differ from the Control, and litter loss in the Fungi treatment did also not differ from the Mix. This result indicates that fungi were the main decomposers in the microcosms. We used two‐way ANOVA to assess litter decomposition differences across microbial treatments and time points. The results suggest that not only the microbial treatment but also time points affected litter loss (Table S11). The mix treatment showed a significantly stronger litter loss rate compared with other treatments (Figure S4), suggesting that bacteria and fungi interactions have dynamic effects on litter decomposition.

### A mixture of bacterial and fungal communities enhances plant growth

**FIGURE 6:**
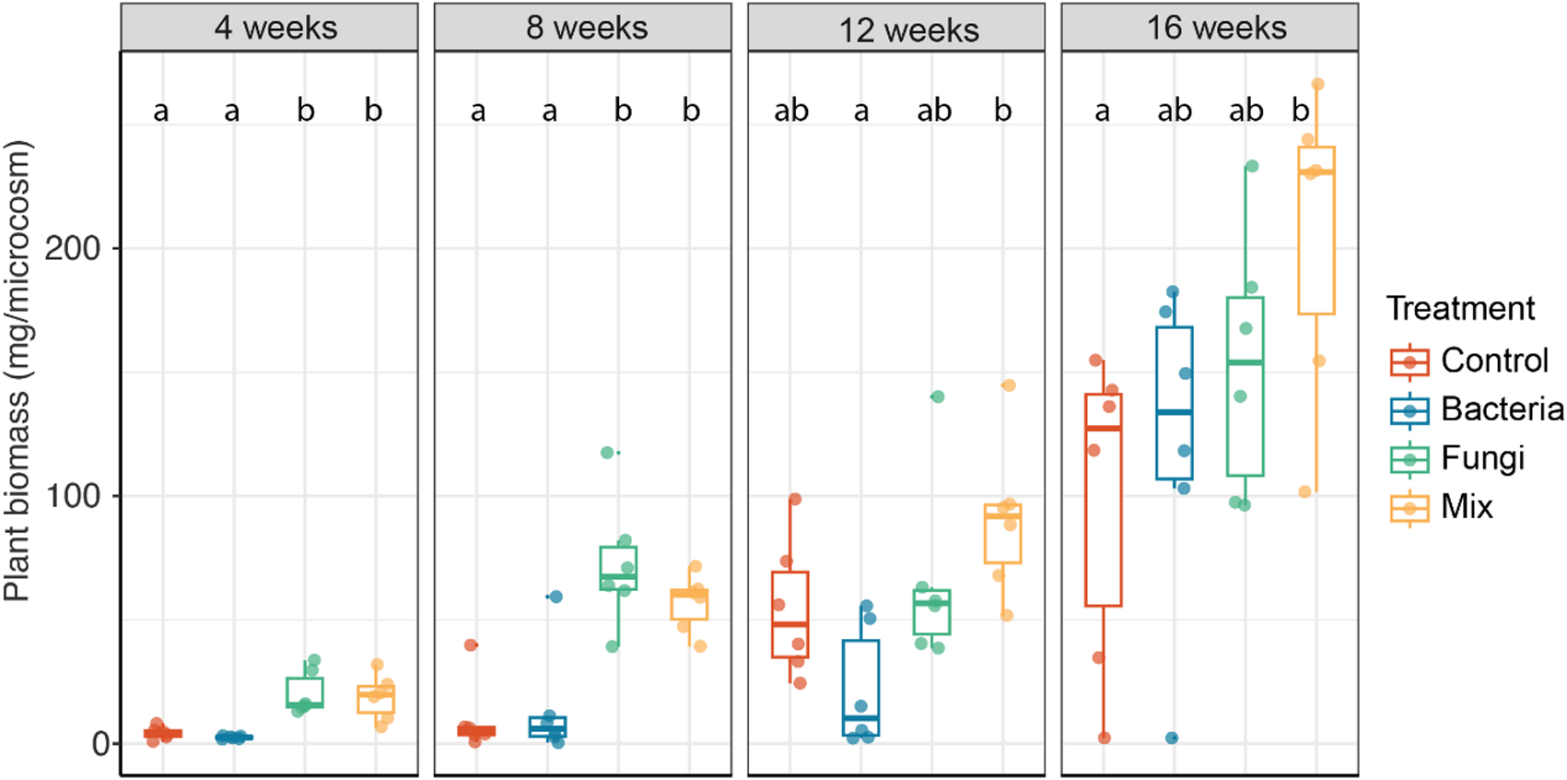
Plant biomass response to microbial treatments. Significance differences are represented by letters (*p* < 0.05, one‐way ANOVA and Tukey HSD test).

The effects of inoculation on plant biomass varied with time (Figure 6 and Table S12). At the 4‐ and 8‐week harvest, inoculation of fungi had significantly enhanced plant biomass (480% and 710% growth stimulation, respectively) compared with the Control or the treatment where only bacteria were inoculated. However, the positive effect of fungal inoculation on plant biomass was no longer observed after 12 and 16 weeks. Interestingly, co‐inoculation of bacteria and fungi resulted in the highest biomass at the final harvest. Additionally, differences in plant biomass between microbial treatments diminished over time. At 16 weeks, the Bacteria and Fungi treatments had a similar plant biomass, while biomass in the Mix treatment was significantly higher than the Control treatment.

## DISCUSSION

### Establishment and rediscovery of the inoculated microbes in the synthetic microbial communities

We found that 60% of the inoculated fungal isolates were rediscovered. However, less than 10% of the inoculated bacterial isolates were rediscovered if we assessed rediscovery based on 100% sequence similarity between inoculated and detected strains. Most of the inoculated isolates were not detected, likely because these microbes were unable to grow or survive in the microcosm. The microcosm was designed to manipulate microbial abundance, providing an environment for studying plant‐microbe interactions. We use calcined clay as a substrate for plant growth in the microcosm. The physicochemical differences between the calcined clay in the microcosms and natural soil likely exerted a selective pressure on the inoculated microbes and probably favoured those that could quickly adapt to the new growth conditions. Alternatively, the bacterial strains were identified by Sanger sequencing, while the microbial communities on litter and root were analysed by Illumina sequencing. The different methods and multiple steps of gene amplification may introduce PCR and sequencing errors. Therefore, some bacterial strains may be rediscovered at lower similarity levels (e.g., 97%).

In this experiment, we observed that a higher diversity of bacteria and fungi colonized plant roots compared with litter. It is well known that plants foster their root microbiome by the exudation of carbon‐rich nutrients, creating microbial populations that are denser on the roots than that of the surrounding soil (Bakker et al., 2020). Moreover, the microbes used in this study were initially isolated from plant roots and are therefore likely better adapted to survive in the rhizosphere than on litter. Note, however, that we also detected many of the inoculated taxa in soil samples (Hartman et al., 2018). Further experimental studies on litter decomposition should specifically include microbes isolated from litter.

We detected various bacteria in the Control treatment, which should have been free of microbes. Thus, it was difficult to reveal to specific effects of bacteria because there is no true control free of bacteria. The autoclaved substrate was checked before it was added to the microcosm, confirming that our sterilization protocol was effective. The plant seedlings were sterilized and pre‐germinated on agar plates. These sterile seedlings with no surrounding microbes were transplanted into the microcosm and the Control treatments were prepared first to prevent cross‐contamination. Thus, the contaminating microbes were most likely introduced after the preparation and assemblage of the microcosms. It is possible that condensation, which formed on gas exchange film at the top of the microcosms, could act as a passage for airborne bacteria to access the microcosms.

Here, we managed to manipulate the presence and richness of fungi. The two microcosms with contamination were removed from the analysis. For bacteria, we also were able to alter bacterial richness. However, contamination occurred in many microcosms and future experiments, perhaps also in more simplified systems without calcined clay, need to be performed to fully manipulate the bacterial treatment. We still feel these data are useful as we were able to manipulate fungal abundance, presence and richness and we could enhance bacterial richness by 2.3–2.6 times from the control to the bacterial treatments.

### Fungi as main drivers of litter decomposition

This study demonstrates that fungi are the main decomposers of plant litter in our experimental system. Litter decomposition in treatments inoculated with fungi was 47% higher compared with the Control treatment or the treatment where only bacteria were inoculated. A number of studies also suggest that bacteria contribute to litter decomposition and produce extracellular enzymes that can degrade lignocellulose, a main component of plant litter (Adhi et al., 1989; Lin et al., 2012). However, we did not observe strong evidence for this. To our surprise, we observed an average of 38.15% litter loss in the Control after the first 4 weeks. This swift litter loss in the Control may have been caused by microbial contaminants that directly consumed available nutrients from litter bags after autoclaving. A mechanistic explanation is missing because we have not assessed the enzymes responsible for litter decomposition or which chemical compounds are degraded first.

Our study is in line with other studies, which identify fungi as the main drivers of litter decomposition probably due to their ability to produce a range of extracellular enzymes (Bugg et al., 2011; Schneider et al., 2012). In this research, although the litter and root harbour have different microbiomes, they still share a considerable number of OTUs between the two sample types. This suggests that most of the isolates used in this experiment can colonize both ecological niches in the microcosms. A microbiome containing more litter or root‐specific colonizers should be included in further studies. However, so far, very few studies have obtained direct experimental evidence for the role of fungi and directly manipulated microbial communities (e.g., bacterial and fungal communities) to investigate the main drivers and identify complementarity. This experiment employed a simplified microbial community with lower bacterial and fungal diversity than natural environments. However, microbial diversity is linked to ecological functions (Wagg et al., 2014; Wagg et al., 2019). Therefore, some microbial functional groups may be absent or underrepresented in our simplified community. To gain a deeper insight into the decomposition process, future research should also examine a wider range of bacterial and fungal communities, as well as protists or soil invertebrates, than the ones used in this study. This is especially important if bacteria or fungi require other microbes to provide specific functions (e.g. if there are complementarity effects among microbes or threshold effects or particular functions are provided by phylogenetically narrow clades of microbes). Moreover, unsterilized field soil or litter can be added as microbial inoculum and compare litter decomposition and plant growth with unsterilized field soil and the added microbial communities.

Interestingly, after 16 weeks, we found that shoot biomass was highest in the Mix treatment pointing to synergistic effects of bacterial and fungal communities. Several studies indicate that bacteria and fungi can complement each other and provide different limiting nutrients to plants resulting in enhanced plant biomass (e.g., (van der Heijden et al., 2015). We also observed the highest microbial richness in the Mix treatment, and this may have contributed to increased plant biomass as observed in earlier works. The investigation of the interkingdom microbial interactions suggests that the bacteria are essential for plant survival and protection against root‐derived filamentous eukaryotes (Durán et al., 2018; Wagg et al., 2014; Wagg et al., 2019). In our case, the fungi probably released more plant‐available nutrients to the surrounding soil, while the bacteria may have benefited plant growth in other ways, for example, by secreting plant growth hormones (Bartoli et al., 2022; Jha & Saraf, 2015).

Of the 35 inoculated fungi, sequences of 9 fungal OTUs (fOTU1, fOTU2, fOTU3, fOTU5, fOTU7, fOTU10, fOTU13, fOTU14 and fOTU21) from 7 genera were found enriched in litter samples and we hypothesize that these fungi likely grew on the litter and contributed most to its decomposition. *Fusarium* is the most abundant genus in litter samples including fOTU1 (*Fusarium solani*), fOTU2 (*Fusarium oxysporum*) and fOTU5 (*Fusarium proliferatum*). Although these and other *Fusarium* spp. are mostly studied for their plant pathogenic lifestyle (Dugan et al., 2003; Ma et al., 2013; Ohara et al., 2003), the plants that received fungal inoculation that included these fOTUs did not exhibit any disease symptoms and plant growth was promoted at the beginning of our experiment. *F. solani, F. oxysporum and F. proliferatum* have been shown to possess moderate lignin‐degrading capacities (Lozovaya et al., 2006; Regalado et al., 1997; Waing et al., 2015) and thus hypothesize that they also promoted litter decomposition in our study. Moreover, *Penicillium* and *Alternaria* spp. have been demonstrated to decompose lignin and cellulose (Song & Fan, 2010). This suggests that *Penicillium* fOTU14 and *Alternaria* fOTU10, which were both enriched in our litter samples, share a similar function as those strains. In a recent study, strains from the abovementioned genera (*Fusarium, Penicillium)* were identified as keystone taxa in the litter decomposition process of three different land use types (Zheng et al., 2021). These keystone strains enhanced microbial complexity and showed high enzyme activities of litter decomposition. We found *Rhizoctonia* fOTU3 enriched in the litter, in contrast to (Ivarson, 1974) who reported that *Rhizoctonia* sp. had low survival ability in litter during 45 months. Additionally, there is no previous evidence of the role of the genera *Zalerion* or *Colletotrichum* in litter decomposition, and it is, therefore, difficult to deduce the contributions of the enriched fOTU7 (*Zalerion* sp.) and fOTU21 (*Colletotrichum* sp.) to the results we observed in our experiment. To further investigate how these enriched fungal OTUs decompose plant litter, metatranscriptomic sequencing could be performed to determine the activity of functional genes involved in decomposition.

### Microbial effects on plant growth

Litter decomposition is an important process for nutrient cycling in natural ecosystems (Floudas et al., 2020; Hättenschwiler et al., 2005; Krishna & Mohan, 2017). During decomposition, the C:N ratio decreases and inorganic nutrients are released into the surrounding environment (Crowther et al., 2012). In our experiment, we saw the strongest litter mass loss during the first 4 weeks in the two treatments inoculated with fungi. The increase of plant growth in microcosm with fungi after 4 and 8 weeks may therefore be related to increased nutrient release from the decomposing material. Moreover, previous studies also suggest that microbes (e.g., fungi) can promote plant growth by exuding plant‐growth‐promoting compounds or liberating (micro) nutrients (Hayat et al., 2010). Further studies need to include a control treatment without litter to assess the potential effects of litter on plant growth. We also noticed two plants were not growing at 16 weeks. These ungrown plants may have different seed properties compared with the other plants (e.g., seed microbiome).

In this study, we isolated bacteria from (*Trifolium pratense*) and tested their effects on plant growth (*P. vulgaris*) and litter decomposition (*Lolium multiflorum*). These plant species all co‐occurred at the FAST experimental field site. This means that ecologically relevant interactions between plants and their microbes are investigated because the plant species and microbial taxa are present at the same location. Yet, different plant species have their preference for their microbiome (Bai et al., 2022). It would be interesting to isolate and test the plant growth and litter decomposition of one plant species in the future.

In conclusion, this study provides experimental evidence that fungi are the main decomposers of plant litter in our microcosms. The root and litter have their preferred microbiome when they are receiving the same microbial treatment. The presence of bacteria and fungi together may benefit plant growth. These findings pave the way for a deeper understanding of fungi and bacteria interactions and community succession during litter decomposition, which could eventually be used to develop microbial solutions to enhance litter decomposition and nutrient cycling in agroecosystems.

## AUTHOR CONTRIBUTIONS

**Changfeng Zhang:** Conceptualization (equal); data curation (equal); formal analysis (equal); investigation (equal); methodology (equal); writing – original draft (equal); writing – review and editing (equal). **Simone de Pasquale:** Data curation (equal); investigation (equal); methodology (equal). **Kyle Hartman:** Resources (lead); writing – review and editing (supporting). **Claire E. Stanley:** Conceptualization (supporting); writing – review and editing (supporting). **Roeland L. Berendsen:** Supervision (supporting); writing – original draft (equal); writing – review and editing (supporting). **Marcel G.A. van der Heijden:** Conceptualization (lead); project administration (lead); supervision (lead); writing – original draft (equal); writing – review and editing (equal).

## CONFLICT OF INTEREST STATEMENT

The authors declare no conflicts of interest.

## Supporting information


**Data S1.** Supporting information.

**Table S1.** Taxonomy of selected bacteria and fungi for creating synthetic communities. (A) Selected bacteria for creating bacterial inoculum. (B) Selected fungi for creating fungal inoculum.

**Table S4.** Taxonomy table of bOTUs.

**Table S5.** Taxonomy table of fOTUs.

## Data Availability

The raw sequencing data were deposited at the European Nucleotide Archive (http://www.ebi.ac.uk/ena) by the study accession PRJE4741.
