# Supplementary material for: The microbial contribution to litter decomposition and plant growth
Source: Environ Microbiol Rep. 2023 Nov 29;16(1):e13205. doi: 10.1111/1758-2229.13205 (PMC10866077; doi:10.1111/1758-2229.13205)
Supplement: Supplementary file 1 — Data S1. Supporting information. [file EMI4-16-e13205-s003.docx]

SUPPLEMENTARY METHOD

## Fungal isolation and identification

Individual plants of *Trifolium pratense* were collected from the Farming Systems and Tillage (FAST) experiment (Wittwer *et al.* 2021) and five separate growth chamber experiments (Hartman 2018). Upon harvest, the plants were removed from their pots and the roots were shaken to remove bulk soil. In addition, naturally collected individuals were excavated from the field with a hand shovel, shaken to remove bulk soil, and placed in a plastic bag. In the lab, all root samples were rinsed with distilled H_2_O to remove the loosely adhering soil particles, and 3-5 mm root fragments were cut from the lateral roots into a dish of sterile distilled H_2_O with a pair of flamed scissors. In a sterile laminar flow cabinet, the root fragments were surface sterilized by agitating in 95% EtOH for 15s, 30% H_2_O_2_ for 15s, and finally two separate rinses in sterile distilled H_2_O.

Three sterilized *Trifolium* root fragments per plate were placed on modified MMA or Malt Extract agar (MEA) (Sigma Aldrich, St.Louis, MO USA) plates amended with 15 μg/mL oxytetracycline (Sigma Aldrich, St. Louis, MO USA) to inhibit bacteria growth. All plates were incubated at 25 ºC until single hyphae were visible on the plate surface. Small fragments of individual hyphae were cut from the plates with a sterilized scalpel and sub-cultured at least three times on MMA or MEA plates. The isolates were subsequently re-plated for PCR-based taxonomy identification (see below) or preserved to create the fungi reference stock. For this, re-plated isolates were allowed to grow until fungal biomass covered the plate. In a sterile laminar flow cabinet, ten plugs of each isolate were punched out from the plate with a flamed cork borer (ø 2.5 mm). Five plugs were placed in a 2 mL cryogenic tube (Thermo Scientific, Waltham, MA, USA) containing 50% glycerol (v/v final) and stored long-term at -80 °C. The other five plugs were placed in a 2 mL cryogenic tube (Thermo Scientific, Waltham, MA, USA) containing sterile distilled H_2_O and stored at room temperature in the dark.

A small amount of fungal biomass from each isolate was scraped from the surface of the agar plate and placed in a sterile 1.5 mL tube. Fungal DNA was extracted with the REDExtract-N-Amp Plant PCR Kit (Sigma Aldrich, St. Louis, MO, USA) following the manufacturer’s instructions. The extracted DNA was used as a template in PCR reactions. Each 20 μL PCR reaction per isolate contained 10 μL REDExtract-N-Amp PCR Ready Mix (Sigma Aldrich, St. Louis, MO, USA), 400 nM of each primer ITS5 and ITS4 (White, Bruns, Lee & Taylor 1990) 4 μL of template DNA, and the remaining volume sterile distilled H2O. All reactions were performed in an iCycler instrument (BioRad, Hercules, CA, USA) with the cycling conditions of 2 min at 95 ℃, followed by 35 cycles of 1 min at 94 ℃, 1 min at 54 ℃ and 1 min at 72 ℃ and a final extension of 10 min at 72 ℃. PCR amplicons were verified on a 1% agarose gel. The reactions were purified and sequenced using the Sanger method with ITS5 as the sequencing primer by Microsynth AG (Balgach, Switzerland).

The resulting AB1 sequencing files were converted into FASTQ file format using EMBOSS v6.6.0(Rice, Longden & Bleasby 2000). Sequences were quality filtered by trimming 50 bp from the 5’ and 3’ ends and then progressively trimming nucleotides from both ends at a mean Phred score <25 (window size 5, step size 2). Finally, sequences <400 bp or with a mean Phred score <30 were discarded. Quality filtering was performed using PRINSEQ v0.20.4 (Schmieder & Edwards 2011). Quality sequences were used for taxonomy assignment using the RDP classifier against the UNITE database v7 (Abarenkov *et al.* 2010) as implemented in QIIME v1.8 (Caporaso *et al.* 2010).

# REFERENCES

Abarenkov K., Henrik Nilsson R., Larsson K.-H., Alexander I.J., Eberhardt U., Erland S., … Kõljalg U. (2010) The UNITE database for molecular identification of fungi – recent updates and future perspectives. *New Phytologist* **186**, 281–285.

Caporaso J.G., Kuczynski J., Stombaugh J., Bittinger K., Bushman F.D., Costello E.K., … Knight R. (2010) QIIME allows analysis of high-throughput community sequencing data. *Nature Methods* **7**, 335–336.

Hartman K.J. (2018) Molecular and experimental approaches for exploring the role of the soil and root microbiome in agroecosystem functioning.

Rice P., Longden I. & Bleasby A. (2000) EMBOSS: the European molecular biology open software suite. *Trends in genetics* **16**, 276–277.

Schmieder R. & Edwards R. (2011) Quality control and preprocessing of metagenomic datasets. *Bioinformatics* **27**, 863–864.

White T.J., Bruns T., Lee S. & Taylor J. (1990) Amplification and direct sequencing of fungal ribosomal RNA genes for phylogenetics. *PCR protocols: a guide to methods and applications* **18**, 315–322.

Wittwer R.A., Franz Bender S., Hartman K., Hydbom S., A Lima R.A., Loaiza V., … A van der Heijden M.G. (2021) Organic and conservation agriculture promote ecosystem multifunctionality. *Science Advance* **7**.

#

# SUPPLEMENTARY FIGURE

**
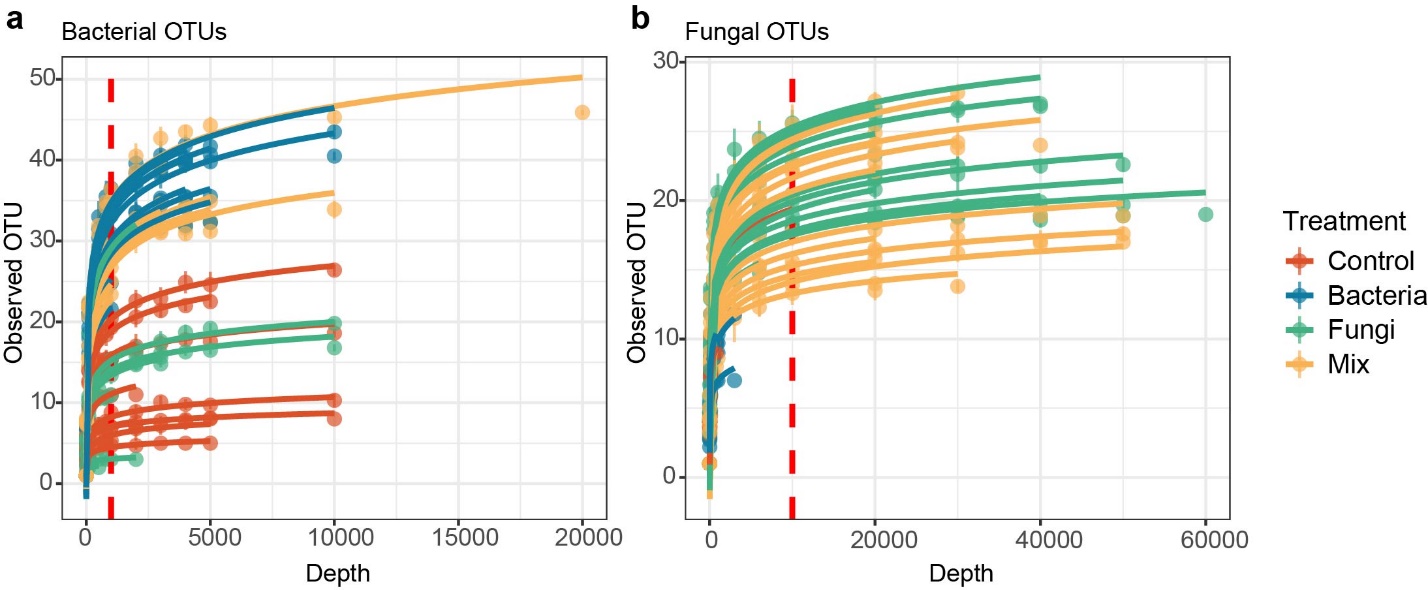
**

Figure S1. Rarefaction cure of bacterial and fungal communities. The microbial treatments are depicted in four colors. The red dash lines indicate the selected rarefaction depth.


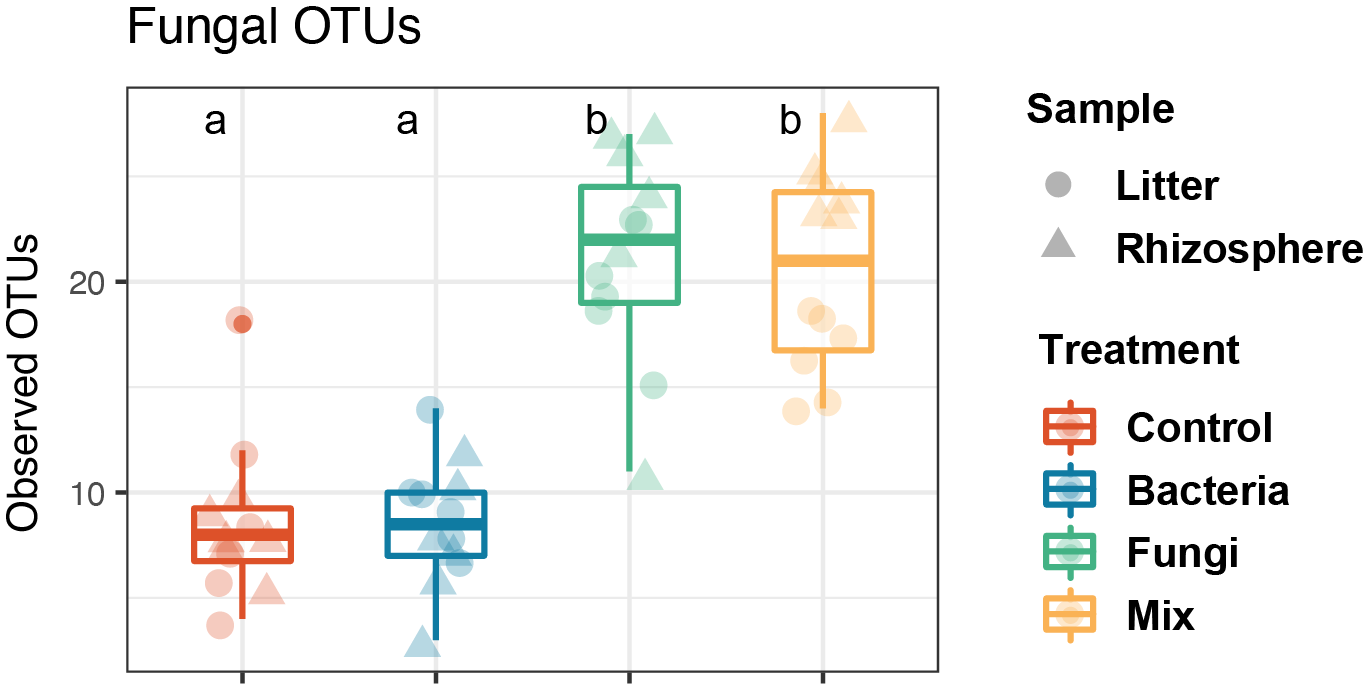


Figure S2. Unrarefied fungal OTU richness. The microbial treatments are depicted by four colors. The sample types are indicated by different symbols.


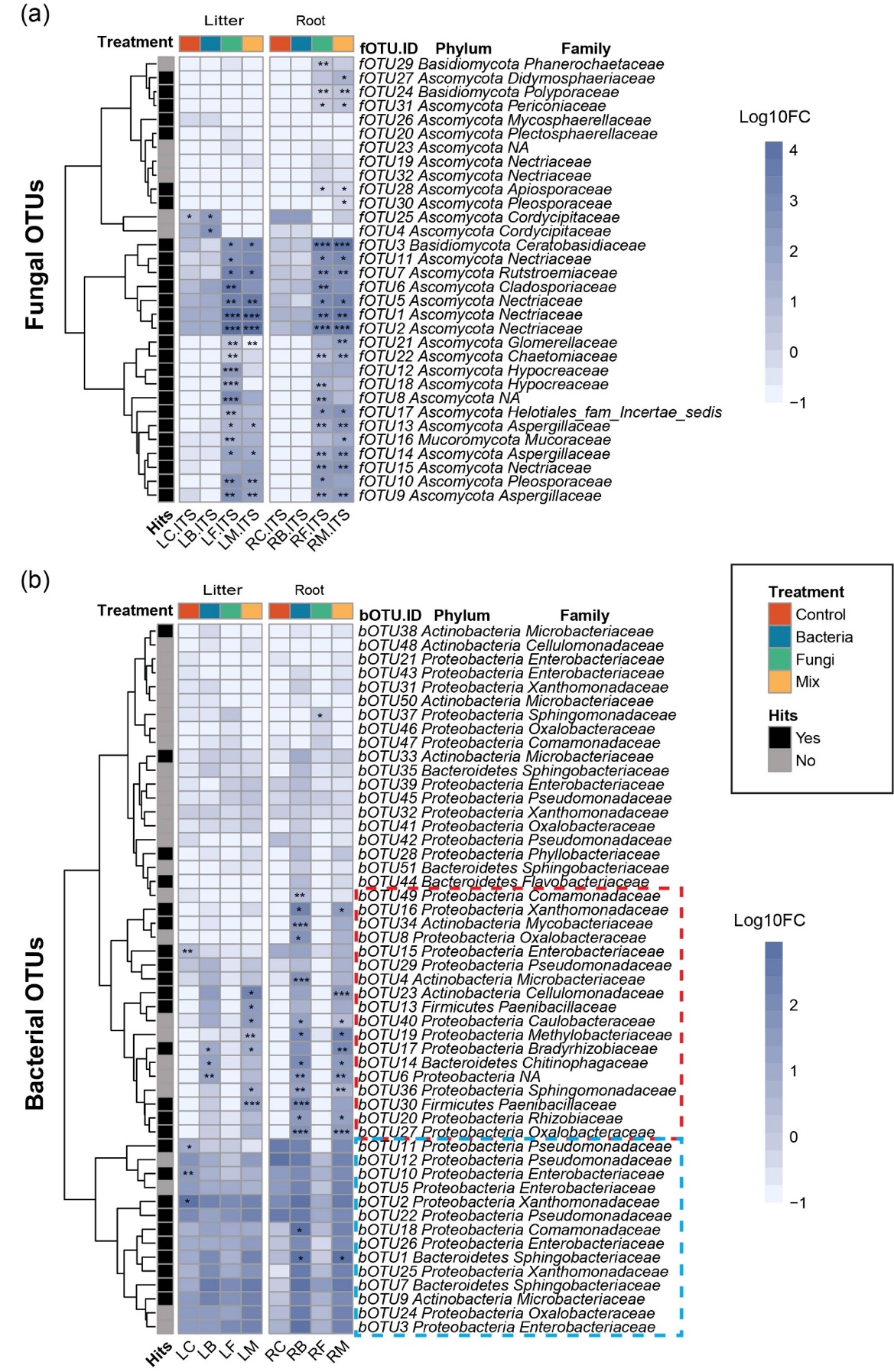


Figure S3. Relative abundance of bacterial and fungal OTUs in litter (L) and root (R) samples inoculated with fungi (F), bacteria (B), fungi and bacteria (M) or non-inoculated controls (C). Black colored boxes (Hits) refer to OTU sequences that are similar to inoculated bacterial or fungal while grey colored boxes (Non-hits) are OTUs that are not similar to the inoculated bacteria and fungi. The asterisk in the heatmap cells indicate OTUs that are significantly and positively correlated to one or more treatments (p* < 0.05, p** < 0.01, p*** < 0.001). (a) Litter and root fungal OTUs. (b) Litter and rhizosphere bacterial OTUs. Only OTUs presents in at least 3 samples are shown here. Red dashed frame indicates the abundant bOTUs of bacterial inoculated treatments of root microbiome. Blue dashed frame indicates bOTUs abundant in all treatments of litter microbiome. The dendrogram is based on hierarchical clustering. Litter, rhizosphere and microbial treatments are represented by different colors.


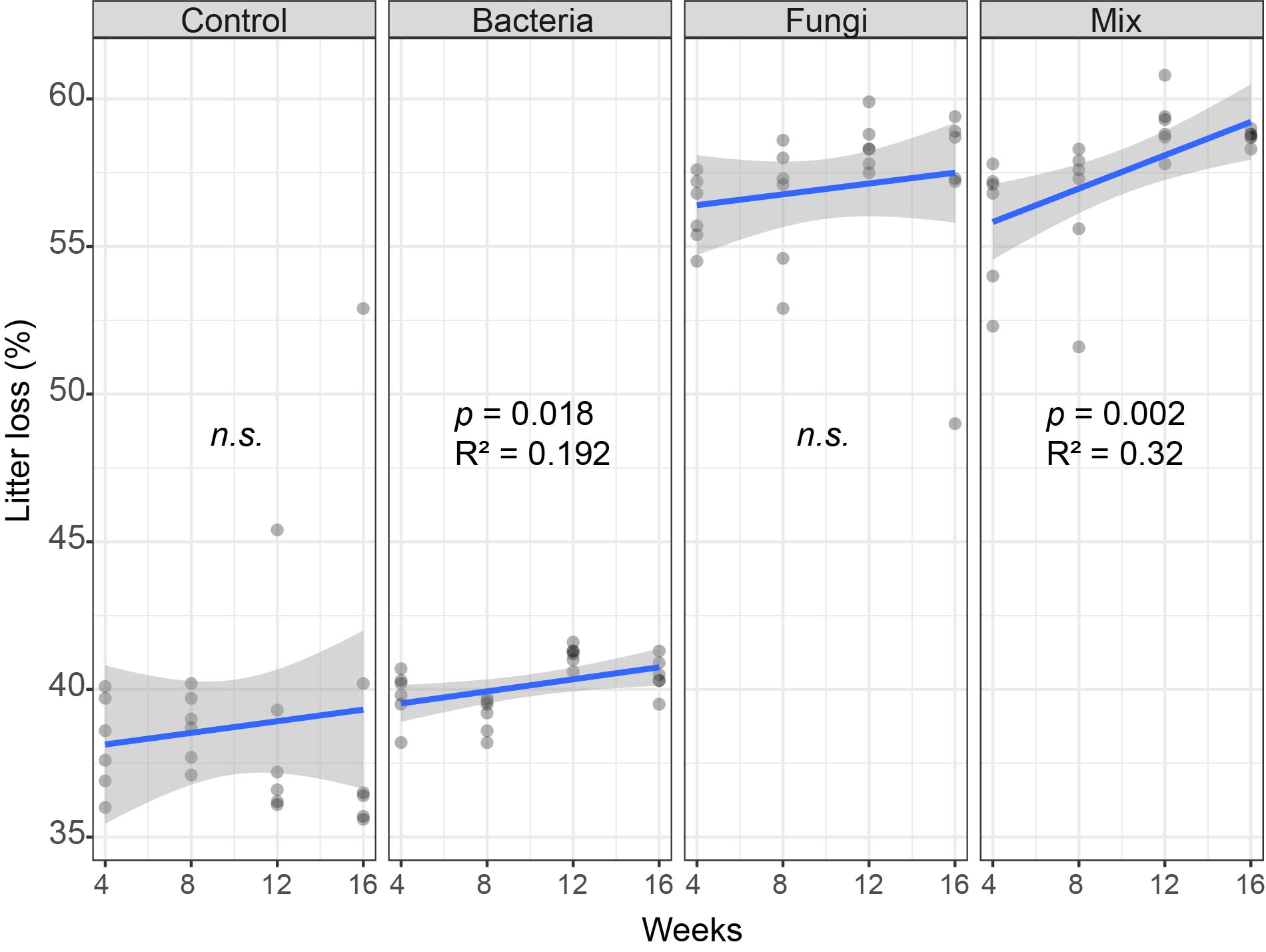


Figure S4. Litter loss increased with time in Bacteria and Mix treatment.

# SUPPLEMENTARY TABLE

**Table S1** **Taxonomy of selected bacteria and fungi for creating synthetic communities.**  (A) Selected bacteria for creating bacterial inoculum. (B) Selected fungi for creating fungal inoculum. (This table is in an excel attachment)

**Table S2** **20ml 15% Hoagland solution added to each microcosm**

| **Macronutrients** | **Concentration (mM)** |
| --- | --- |
| KNO_3_ | 0.9 |
|  |  |
| (NH_4_)H_2_PO_4_ | 0.3 |
| Ca(NO3)2•4H2O | 0.6 |
| MgSO_4_•7H_2_O | 0.15 |
| **Micronutrients** | **Concentration (µM)** |
| KCl | 7.5 |
| H_3_BO_3_ | 3.75 |
| MnSO_4_•H_2_O | 0.3 |
| ZnSO_4_•7H_2_O | 0.3 |
| CuSO_4_•5H_2_O | 0.075 |
| (NH_4_)_6_Mo_7_O_24_•4H_2_O | 0.075 |
| C_10_H_12_FeN_2_NaO_8_ | 3 |

**Table S3 Two-step PCR cycling conditions used to amplify the 16S and ITS sequences.**

| **16S Community Profiling** | | | | | | | **ITS Community Profiling** | | | | | |
| --- | --- | --- | --- | --- | --- | --- | --- | --- | --- | --- | --- | --- |
|  | **First step PCR** | | | **Second step PCR** | | | **First step PCR** | | | **Second step PCR** | | |
|  | Temp. | Time | Cycle | Temp. | Time | Cycle | Temp. | Time | Cycle | Temp. | Time | Cycle |
| 1 | 95℃ | 3 min | 1 | 95℃ | 3 min | 1 | 95℃ | 3 min | 1 | 95℃ | 3 min | 1 |
| 2 | 95℃ | 30 sec | 25 cycles | 95℃ | 30 sec | 10 cycles | 95℃ | 30 sec | 25 cycles | 95℃ | 30 sec | 8 cycles |
| 3 | 75℃ | 10 sec |  | 55℃ | 30 sec |  | 55℃ | 30 sec |  | 55℃ | 30 sec |  |
| 4 | 55℃ | 30 sec |  | 72℃ | 30 sec |  | 72℃ | 30 sec |  | 72℃ | 30 sec |  |
| 5 | 72℃ | 30 sec |  | 72℃ | 5 min | 1 | 72℃ | 5 min | 1 | 72℃ | 5 min | 1 |
| 6 | 72℃ | 5 min | 1 | 10℃ | Hold |  | 10℃ | Hold |  | 10℃ | Hold |  |
| 7 | 10℃ | Hold |  |  |  |  |  |  |  |  |  |  |

**Table S4** **Taxonomy table of bOTUs** (This table is in excel attachment)**.**

**Table S5** **Taxonomy table of fOTUs** (This table is in excel attachment)**.**

**Table S6** **Kruskal Wallis and Dunn’s post-hoc test determine the CFUs differences across microbial treatments.** For bacterial communities, the CFUs were found significant difference among microbial treatments (chi-squared = 23.6, df = 3, p-value < 0.001). For fungal communities, CFUs were found significant difference (chi-squared = 34.8, df = 3, p-value <0.001) among four microbial treatments.

| **Bacterial CFUs** | | **Fungal CFUs** | |
| --- | --- | --- | --- |
| **Comparison** | ***p-*value** | **Comparison** | ***p-*value** |
| Bacteria - Control | 0.001 | Bacteria - Control | 0.416 |
| Bacteria - Fungi | 0.002 | Bacteria - Fungi | < 0.001 |
| Control - Fungi | 0.919 | Control - Fungi | < 0.001 |
| Bacteria - Mix | 1 | Bacteria - Mix | 0.001 |
| Control - Mix | 0.004 | Control - Mix | 0.017 |
| Fungi - Mix | 0.005 | Fungi - Mix | 0.214 |

**Table S7 ANOVA and Tukey HSD test determine the OTU richness differences across microbial treatments.** The bacterial communities were found significantly different among treatments (df = 3, F = 31.94, *p* < 0.001). The fungal communities were found significantly different among treatments (df = 1, F = 35.77, *p* < 0.001)

|  | **Bacteria** | | | | **Fungi** | | | |
| --- | --- | --- | --- | --- | --- | --- | --- | --- |
| **Comparison** | **Mean Difference** | **Lower**  **Bound** | **Upper**  **Bound** | ***p*-value** | **Mean Difference** | **Lower**  **Bound** | **Upper**  **Bound** | ***p*-value** |
| Bacteria-Control | 19.545 | 13.004 | 26.086 | <0.001 | 0.167 | -4.318 | 4.652 | 0.999 |
| Fungi-Control | 2.375 | -4.753 | 9.503 | 0.806 | 12.750 | 8.265 | 17.235 | <0.001 |
| Mix-Control | 17.091 | 10.549 | 23.632 | <0.001 | 12.000 | 7.515 | 16.485 | <0.001 |
| Fungi-Bacteria | -17.170 | -24.298 | -10.042 | <0.001 | 12.583 | 8.098 | 17.068 | <0.001 |
| Mix-Bacteria | -2.455 | -8.995 | 4.087 | 0.745 | 11.833 | 7.348 | 16.318 | <0.001 |
| Mix-Fungi | 14.715 | 7.588 | 21.844 | <0.001 | -0.750 | -5.235 | 3.735 | 0.969 |

**Table S8** **Bacterial community pairwise PERMANOVA results on Bray-Curtis dissimilarities testing the microbial treatment effects and sample type effects.**

| **Factors** | **pseudo-F** | **R^2^** | ***P*-value** |
| --- | --- | --- | --- |
| **Treatment** |  |  |  |
| Control - Bacteria | 7.697 | 0.278 | 0.006 |
| Control - Fungi | 2.449 | 0.126 | 0.306 |
| Control - Mix | 7.577 | 0.275 | 0.006 |
| Bacteria - Fungi | 3.495 | 0.171 | 0.006 |
| Bacteria - Mix | 1.976 | 0.090 | 0.582 |
| Fungi - Mix | 3.809 | 0.183 | 0.006 |
| **Sample type** |  |  |  |
| Litter - Root | 4.197 | 0.097 | 0.001 |

**Table S9 Two-way ANOVA determine the observed OTUs differences across microbial treatments and sample types.** Shown are the treatment, sample type and their interaction effect on observed OTUs.

|  | **Bacterial OTU number** | | | **Fungal OTU number** | | |
| --- | --- | --- | --- | --- | --- | --- |
| **Factor** | **F** | **Df** | ***P*-value** | **F** | **Df** | ***P*-value** |
| Treatment | 35.81 | 3 | <0.001 | 4.36 | 1 | 0.0512 |
| Sample type | 1.45 | 1 | 0.237 | 46.30 | 1 | <0.001 |
| Treatment * Sample type | 2.35 | 3 | 0.091 | 5.99 | 1 | 0.025 |

**Table S10 Fungal community pairwise PERMANOVA results on Bray-Curtis dissimilarities testing the microbial treatment effects and sample type effects.**

| **Factors** | **pseudo-F** | **R^2^** | ***P*-value** |
| --- | --- | --- | --- |
| **Treatment** |  |  |  |
| Fungi - Mix | 1.741 | 0.080 | 0.14 |
| **Part** |  |  |  |
| Litter - Root | 1.039 | 0.438 | 0.001 |

**Table S11 Two-way ANOVA determine the litter differences across microbial treatments and timepoints.** Shown are the treatment, timepoint and their interaction effect on litter loss.

| **Factor** | **F** | **Df** | ***P*-value** |
| --- | --- | --- | --- |
| Treatment | 424.28 | 3 | <0.001 |
| Timepoints | 6.6 | 1 | 0.012 |
| Treatment * Timepoints | 0.69 | 3 | 0.562 |


**Table S12 Two-way ANOVA determine the plant biomass differences across microbial treatments and timepoints.** Shown are the treatment, timepoint and their interaction effect on plant biomass.

| **Factor** | **F** | **Df** | ***P*-value** |
| --- | --- | --- | --- |
| Treatment | 10.48 | 3 | <0.001 |
| Timepoints | 132.08 | 1 | <0.001 |
| Treatment * Timepoints | 2.647 | 3 | 0.054 |
